# Mitomycin C-associated radiofrequency microelectrocautery used in myringotomy in an animal model

**DOI:** 10.1016/S1808-8694(15)30548-6

**Published:** 2015-10-19

**Authors:** Vanessa Chisté Guimarães Faccini, Luiz Lavinsky

**Affiliations:** 1 Master's degree, otorhinolaryngologist; 2 (Federal University of Sao Paulo), professor and researcher at the Medical School, Federal University of Rio Grande do Sul

**Keywords:** mitomycin c, otitis, radiofrequency

## Abstract

This study aimed at describing an alternative surgical technique to the insertion of a ventilation tube in the tympanic membrane: myringotomy by radiofrequency alone and associated with mytomicin C.

**Aim:**

to show a surgical approach that can be simple to execute, not subject to complications arising from the ventilation tube.

**Materials and Methods:**

we compared myringotomy by microknife and by radiofrequency microcautery (0.3 mm and 0.7 mm tips) alone and associated with mytomicin C, considering the time of tympanic closure in Wistar rats. Experimental study.

**Results:**

there was a statistically significant difference between radiofrequency myringotomy and knife myringotomy. As we analyze the radiofrequency approach with the 0.7mm tip associated with mytomicin C (Wilcoxon test), the p value found was lower than 0.001, showing a statistical significance. The maximum tympanic membrane closure time was 44 days and the median found was 14 days.

**Conclusion:**

the radiofrequency myringotomy (with the larger diameter tip) associated with mytomicin C enhances the tympanic membrane healing time.

## INTRODUCTION

Otitis media with effusion (OME) is socially and economically relevant, as this condition is considered the most common cause of hearing loss in childhood and a cause of speech impairment and poor achievement in school. According to the American Academy of Pediatrics and the American Academy of Otolaryngology and Head and Neck Surgery, about 90% of children have OME at any time before entering school, usually between ages 6 months to 4 years. Most of these cases resolved spontaneously within 3 months.^1^

Surgery is indicated in cases where the tympanic cavity effusion does not resolve spontaneously. The main purpose of surgery is to restore middle ear ventilation by eliminating negative intratympanic pressure. Thus, the mucosa is normalized, hair cells increase and the secretion potential is reduced.^2^, ^3^, ^4^

Myringotomy with ventilation tube (VT) placement is the procedure of choice from the moment a patient becomes a candidate for surgery.^1^ This procedure is currently one of the most common surgeries in the US, and is the main reason children undergo general anesthesia.^5^

Inserting VT in the tympanic membrane, however, may cause several complications. Several published studies have described the consequences of placing VT, the main ones being: otorrhea, tympanic membrane perforation following VT extrusion, retraction and tympanic atelectasis, tympanosclerosis, and VT retention for long periods.^6^, ^7^, ^8^, ^9^

Recent studies have demonstrated, by electron microscopy, the presence of biofilms on the surface of VTs. It has been suggested that bacterial clusters, named biofilms, are responsible for chronic otorrhea in patients undergoing tympanostomy with VT placement.^10^, ^11^

Aoki et al. showed that 2 months after tympanic cavity clearance, middle ear effusion and inflammation had regressed and pneumatic space had expanded.^12^

In 1978, Saito et al. initiated studies of electrocautery myringotomies, proving that burns caused by this method resisted healing to a greater extent.^13^

In 1982, Goode undertook the first CO_2_ laser myringotomy in humans.^14^, ^15^ Since then, several studies on this technique have been published.^16^, ^17^, ^18^

In 1994, the biomedical engineering department at our institution developed a device for performing highly precise and controllable electrosurgical microcauterization. Radiofrequency microelectrocautery has several advantages over other methods. The cost of operating this device ranges from 5 to 10% of laser costs. Furthermore, the tip is more accurate because it concentrates all the heat and different angles are possible according to the need. Laser may be limited by the angle of the incident rays.^19^, ^20^

The aim of our study was to evaluate the isolated microelectrocautery radiofrequency myringotomy technique with mitomycin C in an animal model. This technique was compared with a microlancet approach. This study is thus part of a line of research aiming to investigated the various myringotomy techniques that are alternatives to VT insertion.

## METHODS

This study was an experimental comparative trial done using Wistar rats, which was approved by the Institutional Review Board (number 03.173). Trichotomy was done prior to anesthesia for placing the microcautery pad. Anesthesia was attained using intramuscular ketamine (40-90 mg/kg) and xylazine (5-13 mg/kg).

A pilot study had demonstrated appropriate microelectrocautery parameters, as follows: power level 8 (about 30 watts) and temporized operation mode of 1.50 seconds.

The study consisted of 3 groups. The right ear (RE) was the control group in all groups, in which microlancet myringotomy (ML-RE) was undertaken. In group 1 (n = 12), radiofrequency microelectrocautery myringotomy (RF-LE) was carried ou in the left ear (LE) using a 0.3 mm tip. In group 2 (n = 10), RF-LE was carried out with a 0.7 mm tip.

In group 3 (n = 15), RF-LE was carried out with a 0.7 mm tip, and associated with mitomycin C (RFMIT-LE). Following microcautery myringotomy, a small mitomycin C-soaked piece of gelfoam was placed for 10 minutes over the surgical wound and thereafter removed. The mitomycin C concentration was 0.4 mg/dl.

Procedures were documented and recorded with a video camera attached to the surgical microscope (DF Vasconcelos). Follow-up examinations were done every 4 days, and concluded upon healing (closure) of the myringotomy. The study was evaluated binomially, that is, myringotomies were either “open” or “closed.” Myringotomy diameters were not measured in the days following the procedure.

### Statistical Analysis

The median and interquartile range variables were described in the statistical analysis. The Wilcoxon test was applied for comparing right and left ears in each group. The Kruskal-Wallis test was applied for analyzing statistical differences among the left ears in the three groups. Due to variable asymmetry, rank transformation of the frequency values was done, after which the Tukey test was applied to detect differences among groups. The significance level was 5%.

### Calculation of the sample size

Eleven rats in each group were needed for a difference of 1.5 standard deviations among the means of different techniques (microlancet and radiofrequency myringotomy with or without mitomycin C) to be detected with a 90% statistical power and a 0.05 significance level.

## RESULTS

Analysis of the ML-RE in 22 rats in groups 1 and 2 revealed that there was full healing in the entire sample within the first 3 days after the procedure. Results of the RF-LE technique, however, were more asymmetrical. In group 1 (RF-LE – 0.3 mm tip), the median was 5 days (P25 = 3 days, P75 = 7 days). In group 2 (RF-LE – 0.7 mm tip), the calculated median was 10 days (P25 = 7 days, P75 = 14).

In group 3 (ML-RE), the calculated median was 7 days (P25 = 3 days, P75 = 7 days). The median calculated for the RFMIT-LE technique was 14 days (P25 = 10 days, P75 = 30 days).

For groups 1 and 2, we analyzed statistically the right and left ears by applying the Wilcoxon test. The P value in the procedure was 0.023 mm with the 0.3 tip, and 0.007 with the 0.7 mm tip. A comparison of the RF-OE technique in the two groups (0.3 and 0.7 mm tip) applying the Tukey test resulted in P = 0.017.

The analysis of ML-RE with RFMIT-LE in the 15-rat sample in group 3 applying the Wilcoxon test found that P was lower, at 0.001. Comparing RFMIT-LE with RF-LE (0.3 mm tip) yielded a P value below 0.001; a comparison with the RF-LE (0.7 mm tip) yielded a P value equal to 0.004 (Tukey test).

## DISCUSSION

Alternatives to VT insertion have been developed to decrease complications associated with this procedure. Several studies have been published, most of which have used the laser technique as an alternative method. Recent studies described below, however, have shown that radiofrequency as a surgical procedure has the same efficacy in terms of myringotomy patency time, as well as a number of advantages over the laser.

Aoki et al. concluded that 2 months after treatment with VT insertion in the tympanic membrane, the middle ear effusion has disappeared, inflammation in the tympanic cavity had regressed, and the pneumatic area had expanded.^12^ This study supports the theory that 2 months of tympanic cavity clearance are enough for normalizing middle ear function. In our study, therefore, we decided to seek for a simpler myringotomy method that would implement and maintain tympanic patency for an approximately 2-month period.

In our study, we used a radiofrequency technique (0.7 mm tip), and attained longer myringotomy patency. Both techniques (radiofrequency myringotomy with 0.7 mm and 0.3 mm tips) showed a statistically significant difference compared to microlancet myringotomy. The maximum healing time was 21 days (0.7 mm tip); the median in this group was 10 days.

Wang et al. found no correlation between the perforation size and healing time of tympanic perforation.^21^ Our results diverge from these findings, as statistically significant longer healing times were observed when using a 0.7 mm tip compared to a 0.3 mm tip (P = 0.017).

In our study, we attempted to increase the time before myringotomy closure by undertaking this procedure with 0.7 mm tip radiofrequency microcauteries and associating mitomycin C. This substance is an aminoglycoside antibiotic with anticancer properties; it is able to interrupt DNA replication by inhibiting mitosis and protein synthesis. Topical mitomycin C may inhibit fibroblast proliferation in the tympanic membrane, which would delay healing.^22^

Ragab et al. (2002 to 2004) studied 120 ears of children (1-12 years, mean age 5 years) with a diagnosis of OME. These patients were randomized as folllows: radiofrequency myringotomy and radiofrequency myringotomy with mitomycin. These authors found a mean 5.3 week closure time with myringotomy associated with mitomycin C. Using radiofrequency only resulted in a mean 3.5 week closure time. The authors concluded that mitomycin C prolongs the myringotomy patency time and stressed that the effectiveness of this substance appears to be increased in the presence of middle ear inflammation.^23^

Lachan et al. (2005) published an experimental study in rabbits to compare radiofrequency only with radiofrequency associated with mitomycin C. The group undergoing myringotomy associated with mitomycin C had a mean 5.45 week patency, compared to the radiofrequency technique only, wit a mean 1.60 weeks. The authors concluded that mitomycin C is an effective method to extend the myringotomy patency time.^24^

**Table 1:** Comparison of myringotomy healing time among groups.

			Days - median (interquartile range P25-75)			
Ear	Group ** (n=12)		Group 2** (n=10)		Group 3** (n=15)	
Right (control)	3 (3 a 3)	P*=0.023	3 (3 a 3)	P*=0,007	7 (3 a 7)	P* <0,001
Left	5 (3 a 7)		10 (7 a 14)		14 (10 a 30)	

* Comparison among groups of left and right ears (sample and control ear) - Wilcoxon test

** Comparison of left ears among groups: group 1 vs. Group 2 - P = 0.012, group 1 vs. Group 3 - P <0.001, vs group 2. Group 3 - P = 0.004 Source: The Author (2007)

These authors carried a later experimental study in rabbits to compare laser and radiofrequency myringotomy only or associated with mitomycin C. This study showed no difference in healing time between both techniques isolatedly. However, associating mitomycin C prolonged the patency similarly in both techniques. The authors pointed out that since there was no statistically significant difference between both procedures (CO_2_ laser and radiofrequency), radiofrequency could be an interesting alternative to laser, due to its safety, low cost and ease of handling. Associating mitomycin C, in their study, helped prolong the myringotomy patency time.^25^

Wenzel et al. studied radiofrequency myringotomy without mitomycin C in 83 children with OME. Results were quite satisfactory, the average myringotomy healing time being 2.83 months (standard deviation = 1.39).^26^

Radiofrequency myringotomy (0.7 mm tip) associated with mitomycin C, as presented in our study, demonstrated a more statistically significant increase compared to the first two study groups (radiofrequency 0.7 mm and 0.3 mm tips); the maximum tympanic membrane healing time was 44 days (the median was 14 days).

Our study results diverged from those presented in the four studies above on radiofrequency myringotomy. These results may be due to the fact that Wenzel et al.'s 26 and Ragab et al.'s ^23^, studies carried out myringotomy in children with middle ear effusion. MIddle ear inflammation appears to prolong patency. Additionally, the myringotomy diameter in their subjects was larger than in the animals in our study.

The other 2 studies by Lachanas et al.^24^, ^25^ used rabbits, whereas we used Wistar rats. Moreover, the radiofrequency current power and the device were different in our study. The maximum power of our device is 35 watts; we used the power 8 setting (operation mode timed in 1.5 seconds) for obtaining about 30 Watts. Lachanas et al. did not report exposure times to the radiofrequency current on the tympanic membrane. These factors may have contributed to lower myringotomy closure times in our study.

Jassir et al. showed a dose-response curve for the topical application of mitomycin C; a 0.4 mg/ml dose resulted in a longer patency time compared to 0.05 mg/ml and 0.2mg/ml. However, a 2.0 mg/ml dose did not increase the patency, and was associated in most cases with otorrhea. Otoacoustic emissions were carried out after the procedure and revealed no cochlear damage.^27^, ^28^

Jang et al., in a study of the effects of mitomycin C on a tympanic membrane fibroblast culture, concluded that the most significant antiproliferative effect was attained at a 0.4 mg/ml concentration applied during 10 minutes.^22^

As a result of these studies, we adopted what was considered the most effective dose and duration of use of mitomycin C: 0.4mg/ml during 10 minutes.

In our study, mitomycin C did not trigger otorrhea in our sample animals. We did not investigate cochlear function after mitomycin use.

Our results showed that patency after radiofrequency myringotomy was more protracted than patency following microlancet myringotomy. There was also a significant increase in patency when mitomycin C was associated, showing that this technique may be an optional and effective method for myringotomy with VT insertion.

Radiofrequency myringotomy has a number of advantages over laser myringotomy, namely lower cost, simpler handling, heat is concentrated in the tip resulting in increased accuracy, and the tip may be angled as required. Thus, microcauteries are superior to laser in that the latter may be restricted depending on the angle of incident rays.^20^

It is important to emphasize that our study was conducted in rats in which the tympanic membrane diameter is much smaller than that in humans; hence, myringotomy diameters were less than those typically done in humans. Furthermore, our study was done in ears without active otitis, that is, with no middle ear mucosa inflammation. Consequently, myringotomy closure times may have been lower, since the presence of a pathological process in the middle ear interferes with the quantitative analysis of the patency period. Future studies may confirm this hypothesis, by causing otitis media with effusion in experimental animals.

## CONCLUSION

This study demonstrated that radiofrequency microelectrocautery myringotomy showed a statistically significant difference in myringotomy healing time compared to microlancet myringotomy. Mitomycin C associated with the radiofrequency myringotomy (0.7 mm tip) method increased the patency period, with statistically significant results (P <0.01).
